# Bacterial β-glucosidase function and metabolic activity depend on soil management in semiarid rainfed agriculture

**DOI:** 10.1002/ece3.88

**Published:** 2012-04

**Authors:** Rosa Cañizares, Beatriz Moreno, Emilio Benitez

**Affiliations:** Department of Environmental Protection, Estación Experimental del Zaidín (EEZ), CSICProfesor Albareda 1, 18008 Granada, Spain

**Keywords:** Ecosystem services, genomics, β-glucosidase, rainfed farming, semiarid climate, transcriptomics

## Abstract

Genomic and transcriptomic approaches were used to gain insights into the relationship between soil management and bacterial-mediated functions in an olive orchard agroecosystem. Four management practices were assessed in a 30-year trial in a semiarid Mediterranean region. Transcriptional activity of bacterial 16S rRNA genes increased in noncovered soils, indicating higher microbial maintenance requirements to thrive in less favorable environmental conditions. The 16S rRNA transcript:gene copy ratio confirmed this assumption and pointed toward a much higher constitutive expression from rRNA operons in noncovered soils and to even higher expression levels when spontaneous vegetation was removed chemically. As described for 16S rRNA, potential transcription did not reveal the real transcription of bacterial β-glucosidase genes, and higher gene expression in noncovered soils plus herbicides was evidenced. Since no relationship between total or soluble organic carbon and bacterial β-glucosidase transcription was found, the above hypothesis could indicate either that soluble organic carbon is not the main pool of enzyme-inducing substrates or that constitutive production of bacterial β-glucosidase enzymes increases as soil conditions worsen.

## Introduction

In rainfed agriculture, the management of spontaneous vegetation is often the only input of organic carbon into agricultural soils of the Mediterranean area. Low organic carbon pool, as a result of high mineralization rates and low biomass production during dry periods (Borken and Matzner 2009), characterizes most of the agriculture soils of the southern Mediterranean region. The systematic use of tillage or agrochemicals under these climatic conditions intensifies soil organic carbon (SOC) depletion (Masciandaro and Ceccanti 1999; Evrendilek et al. 2004) and consequently soil physical, chemical, and biological processes (Benitez et al. 2006; Gómez et al. 2009). In this scenario, the identification of practices to ensure sustainable management of soil resources is critical. The target should be to enhance soil resilience and to improve ecosystem services (Lal 2009). Because of their key role in many ecosystem functions (Singh et al. 2010), soil microorganisms form the basis of the ecosystems on which agriculture and food production depend. The response of the soil microbial community to different agriculture practices may therefore determine soil stability and sustainability, especially under fluctuating climatic conditions and in fragile ecosystems, as in Mediterranean areas.

Dynamics and mineralization of organic carbon constitute one of the many bacterial-mediated processes of vital importance in soils. Soil bacteria play a central role in the global carbon cycle, participating in the sequestration of carbon in soil organic matter as well as in the release of carbon dioxide to the atmosphere. Therefore, to establish the relationship between soil management and bacterial-mediated functions related to the carbon cycle is a complex task that is necessary in order to evaluate agricultural sustainability.

Molecular analyses help to determine various aspects of the microbial composition and it function in soils (Torvisk and Øvreås 2002). Genomic and transcriptomic approaches have provided information on the effect of management in relation to soil microbial structure and functions (Hirsch et al. 2010; Sofo et al. 2010).

In the present paper, molecular approaches are used to quantify both genetic potential and real transcription of both bacterial ribosomal RNA and β-glucosidase genes with the aim of gaining insights concerning the response to the soil bacterial community after four long-term management practices in semiarid rainfed olive orchards.

## Material and Methods

### Study area and soil sampling

Soil samples were collected in Jaen (southeastern Spain). The main characteristics of the experiment and soils are described in detail in Castro et al. (2008) and Moreno et al. (2009). The study was conducted by designing a random block of four treatments and four replicates (plots). Each plot consisted of 16 olive trees: the central four were controlled and the rest constituted guard rows. The treatments tested were:

-Tillage (T): Three to four annual passes with a disk harrow (at 30-cm deep) and/or a cultivator in spring, followed by a tine harrow in summer.
2. Nontillage and no-cover (NC): Spontaneous vegetation was eliminated by applying the pre-emergence herbicides simazine and diuron in autumn. These had been replaced by oxyfluorfen for five years until the present. In the spring, glyphosate was applied locally.
3. Cover crop plus herbicides (CH): Weeds were left to grow each year to be eliminated in March with herbicides. Initially, diquat/ paraquat was used before being replaced at a later date by glyphosate.
-Cover crops plus mower (CM): Weeds were eliminated with various passes of a chain mower at the end of spring, usually when the plants had completed or almost completed their vegetative cycle.

Two samples were collected in the center of each plot by a modified soil sample ring kit (Eijkelkamp Agrisearch Equipment, Giesbeek, Netherlands) that includes a cylinder of 20 cm (depth of sampling) specifically manufactured for this purpose and then bulked. The bryophyte layer was eliminated (when present) in the CH and CM treatment and weeds were cut to ground level. For the isolation of nucleic acids, subsamples of fresh soil were immediately frozen in liquid nitrogen. The samples were stored at –80°C until molecular analyses were made.

### Chemical analysis

SOC was determined using the Walkley–Black wet dichromate oxidation method (M.A.P.A. 1986). Water-soluble carbon (WSC) was extracted and determined as described in Benitez et al. (1999).

### Nucleic acid isolation from soils and cDNA synthesis

For each soil sample replicate, total DNA was separately extracted from four 1 g subsamples by the bead-beating method, following the manufacturer's instructions for the MoBio UltraClean Soil DNA Isolation kit (MoBio laboratories, Solana Beach, CA, USA). The extracts were pooled and further concentrated at 35°C with a Savant Speedvac® concentrator to a final volume of 20 µl.

Total RNA was extracted from four 2 g subsamples of each replicate following the manufacturer's instructions MoBio RNA PowerSoil Total RNA Isolation kit (MoBio laboratories, Solana Beach, CA, USA). For the removal of residual DNA, DNase I enzyme was added using Roche RNase-Free DNase Set (Roche Applied Science, Penzberg, Germany) following the manufacturer's instructions. The extracts were pooled and further concentrated at 35°C with a Savant Speedvac® concentrator (Thermo Fisher Scientific, Waltham, MA, USA) to a final volume of 80 µl. The cDNA was synthesized from 1 to 2 µg of total RNA-DNase using Transcriptor High Fidelity cDNA Synthesis Kit according to the manufacturer's instructions (Roche Applied Science, Penzberg, Germany). The synthesis reaction was carried out at 50°C for 30 min. The concentration and quality of the final DNA/RNA/cDNA samples were checked using Nanodrop® ND-100 spectrometer (Nanodrop Technologies, Wilmington, DE, USA).

### Real-time PCR assays

Real-time PCR was performed to quantify 16S rRNA and β-glucosidase gene and transcript copy numbers both in soil DNA and in cDNA extracts. The 16S rRNA genes were determined with the universal primers for V3 hypervariable region of 16S rRNA eubacteria P1 and P2 (Muyzer et al. 1993), and the β-glucosidase gene copies were determined with a set of degenerated primers βgluF2/βgluR4 for conserved motifs of soil bacterial β-glucosidase genes (Cañizares et al. 2011). Each 21 µl of PCR reaction contained 3–8 ng of the DNA or 70–230 ng of the /cDNA, 400 nM of each primer, and 10.5 µl 2x IQ SYBER Green Supermix (Bio-Rad, Munich, Germany). Both were amplified with the real-time PCR program previously described by Cañizares et al. (2011). Two different standard curves were generated using a recombinant plasmid containing one copy of the 16S rRNA fragment and a plasmid recombinant containing one copy of β-glucosidase gene from soil bacteria. The curves were drawn according to Moreno et al. (2009). The relationship between threshold cycle (Ct) and the target gene copy number, and the copy numbers of the real-time standard were calculated as described Qian et al. (2007). Three real-time PCRs were carried out for each DNA/cDNA sample, with the Ct determined in triplicate. Both the 16S rRNA and β-glucosidase gene copy number and expression were quantified on an iQ5 thermocycler using iQ5-Cycler software (Bio-Rad, Munich, Germany).

### Data analyses

Results are the means of 12 replicates (three per plot). The results were submitted to a factorial analysis of variance (ANOVA) using the program STATISTICA (StatSoft Inc., Tulsa, OK). Post hoc Tukey's HSD tests in a one-way ANOVA were employed. *P*-values lower than 0.05 were considered evidence for statistical significance.

## Results and Discussion

**Figure 1:**
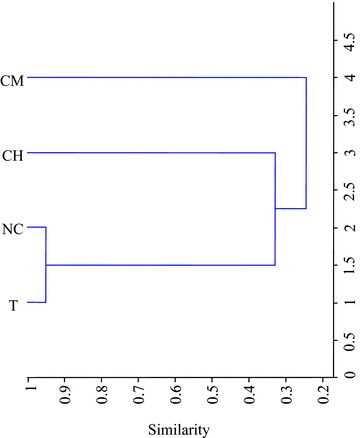
Raup and Crick probability-based index of similarity cluster analyses for DGGE profiles for tillage (T), nontillage and no-cover (NC), cover crop plus herbicides (CH), and cover crops plus mower (CM) soils (adapted from Moreno et al. 2009)

**Figure 2:**
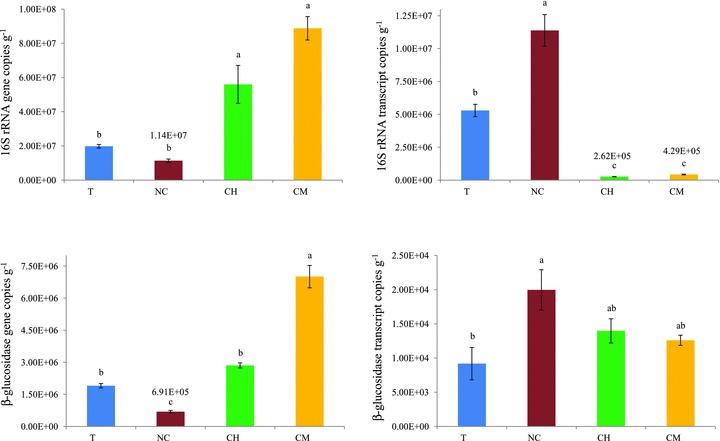
Molecular estimates for tillage (T), nontillage and no-cover (NC), cover crop plus herbicides (CH), and cover crops plus mower (CM) soils. Values are indicated when lower than the first *y* -chart. For each parameter, significant differences are indicated by different letters (*P* < 0.05, ANOVA, Tukey post hoc)

In a previous paper, the authors reported that, after 30 years of the current experiment, the bacterial community structure and diversity are being strongly influenced by soil management (Fig. 1). The positive effects of cover crops on bacterial biomass were also evidenced (Moreno et al. 2009). However, it has been suggested that bacterial biomass should not be used as the only predictor of bacterial activity when comparing soils with different community structures and levels of physiological stress, because of the influence of these factors on specific activity (Stromberger et al. 2011). To provide a valid description of metabolically active members of the soil-bacterial community, analyses of transcriptionally active genes are required (Girvan et al. 2003). In fact, a different trend was revealed when 16S rRNA transcripts were considered (Fig. 2). Covered soils displayed the lowest bacterial constitutive transcriptional activity, indicating a lack of relationship between soil bacterial activity and biomass, as previously reported for other soil conditions (Stromberger et al. 2011).

The ratio between 16S rRNA transcript to gene copies indicate a much higher constitutive expression from rRNA operons in soils without cover; even higher when weeds were eliminated by chemical methods (Table 1). It has been demonstrated that bacteria under environmental stress can maintain an elevated background pool of mRNA, providing an adaptation for rapid onset of degradation as soon as substrates again become available (Nicolaisen et al. 2008), but, why also of ribosomal RNA? This excess transcription capacity could indicate a risky metabolic expense under conditions of low nutrient availability and drought (Klappenbach et al. 2000). Considering that the bulk of the ribosome pool is not required for protein synthesis (Fegatella et al. 1998), the continuous metabolic activity of bare-soil bacteria, even under unfavorable conditions, could suggest some mechanism of adapting to environmental stress. The ability of soil bacteria to acclimate to stress by changing their allocation resource and growth into survival has been previously reported (Schimel et al. 2007), and the high metabolic activity could therefore be a sign of the capacity of the bacterial community to respond to an ecosystem-level stressor (drought) under unfavorable soil conditions.

**Table 1:** Soil Organic Carbon (SOC), Water Soluble Carbon (WSC), transcript:gene copy ratios in tillage (T), nontillage and no-cover (NC), cover crop plus herbicides (CH), and cover crops plus mower (CM) soils.

	SOC (g kg^–1^)	WSC (μg g^–1^)	16S rRNA transcripts:gene	β-glucosidase transcripts:gene
T	9.4 ± 3.1 a	31.3 ± 5.58 c	0.268	0.005
NC	4.6 ± 0.7 b	39.8 ± 6.48 c	1.002	0.029
CH	6.8 ± 3.2 ab	105 ± 16. 5 a	0.005	0.005
CM	8.3 ± 2.0 a	72.6 ± 12.0 b	0.005	0.002

For each parameter, significant differences are indicated by different letters (*P* < 0.05, ANOVA, Tukey post hoc).

Soils under cover crops plus mower displayed the highest genetic potential to produce bacterial β-glucosidase enzyme (Fig. 2). Cover crops plus herbicides and tillage managements shared the same number of gene copies, whereas the lowest values were detected in noncovered plus herbicides soils. As described above for 16S rRNA, the potential transcription did not reveal the real transcription of β-glucosidase genes. The β-glucosidase transcript:gene copy ratio indicates higher expression in soils without cover plus herbicides and similar levels for the rest of the treatments (Table 1). In addition, no relationship between available organic carbon (WSC, Table 1) and β-glucosidase transcription or expression was found, although β-glucosidase production is inducible in soils (Burns 1982) and its activity depends on SOC and WSC availability (Pajares et al. 2011). This fact could indicate that constitutive production of bacterial β-glucosidase enzyme, aimed to detect the presence of a potential C substrate (Allison et al. 2011), increases as soil conditions worsen (lower organic matter content and higher metabolic expense), or may be that WSC do not reflect the main pool of enzyme-inducing substrates.

The above assumptions could be strengthened by determining the in situ levels of β-glucosidase enzyme. Soil proteomics will undoubtedly help to solve discrepancies in genetic potential and apparent functions associated with enzyme production, although many improvements are still needed (Nannipieri 2006; Cañizares et al. 2011).

## References

[b1] Allison SD, Weintraub MN, Gartner TB, Waldrop MP, Shukla G, Varma A (2011). Evolutionary-economic principles as regulators of soil enzyme production and ecosystem function. Soil Enzymology.

[b2] Benitez E, Nogales R, Elvira C, Masciandaro G, Ceccanti B (1999). Enzyme activities as indicators of the stabilization of sewage sludges composting with Eisenia foetida. Bioresource Technol.

[b3] Benitez E, Nogales R, Campos M, Ruano F (2006). Biochemical variability of olive-orchard soils under different management systems. Appl. Soil Ecol.

[b4] Borken W, Matzner E (2009). Reappraisal of drying and wetting effects on C and N mineralization and fluxes in soils. Glob. Change Biol.

[b5] Burns RG (1982). Enzyme activity in soils: Location and a possible role in microbial ecology. Soil Biol. Biochem.

[b6] Castro J, Fernández-Ondoño E, Rodríguez C, Lallena AM, Sierra M, Aguilar J (2008). Effects of different olive-grove management systems on the organic carbon and nitrogen content of the soil in Jaén (Spain). Soil Tillage Res.

[b7] Cañizares R, Benitez E, Ogunseitan OA (2011). Molecular analyses of [beta]-glucosidase diversity and function in soil. Eur. J. Soil Biol.

[b8] Evrendilek F, Celik I, Kilic S (2004). Changes in soil organic carbon and other physical soil properties along adjacent Mediterranean forest, grassland, and cropland ecosystems in Turkey. J. Arid Environ.

[b9] Fegatella F, Lim J, Kjelleberg S, Cavicchioli R (1998). Implications of rRNA operon copy number and ribosome content in the marine oligotrophic ultramicrobacterium sphingomonas sp. strain RB2256. Appl. Environ. Microbiol.

[b10] Girvan MS, Bullimore J, Pretty JN, Osborn AM, Ball AS (2003). Soil type is the primary determinant of the composition of the total and active bacterial communities in arable soils. Appl. Environ. Microbiol.

[b11] Gómez JA, Álvarez S, Soriano MA (2009). Development of a soil degradation assessment tool for organic olive groves in southern Spain. CATENA.

[b12] Hirsch PR, Mauchline TH, Clark IM (2010). Culture-independent molecular techniques for soil microbial ecology. Soil Biol. Biochem.

[b13] Klappenbach JA, Dunbar JM, Schmidt TM (2000). rRNA operon copy number reflects ecological strategies of bacteria. Appl. Environ. Microbiol.

[b14] Lal R, Lichtfouse E, Navarrete M, Debaeke P, Véronique S, Alberola C (2009). Laws of sustainable soil management. Sustainable Agriculture.

[b15] M.A.P.A (1986). Métodos oficiales de análisis. Tomo III. Plantas, productos orgánicos, fertilizantes, suelos, agua, fertilizantes orgánicos.

[b16] Masciandaro G, Ceccanti B (1999). Assessing soil quality in different agro-ecosystems through biochemical and chemico-structural properties of humic substances. Soil Tillage Res.

[b17] Moreno B, Garcia-Rodriguez S, Cañizares R, Castro J, Benítez E (2009). Rainfed olive farming in south-eastern Spain: Long-term effect of soil management on biological indicators of soil quality. Agri. Ecosyst. Environ.

[b18] Muyzer G, Waal ECDe, Uitterlinden AG (1993). Profiling of complex microbial populations by denaturing gradient gel electrophoresis analysis of polymerase chain reaction- amplified genes coding for 16S rRNA. Appl. Environ. Microbiol.

[b19] Nannipieri P, Nannipieri P, Smalla K (2006). Role of Stabilised Enzymes in Microbial Ecology and Enzyme Extraction from Soil with Potential Applications in Soil Proteomics. Nucleic acids and proteins in soil.

[b20] Nicolaisen MH, Bælum J, Jacobsen CS, Sørensen J (2008). Transcription dynamics of the functional tfdA gene during MCPA herbicide degradation by Cupriavidus necator AEO106 (pRO101) in agricultural soil. Environ. Microbiol.

[b21] Pajares S, Gallardo JF, Masciandaro G, Ceccanti B, Etchevers JD (2011). Enzyme activity as an indicator of soil quality changes in degraded cultivated Acrisols in the Mexican Trans-volcanic Belt. Land Degrad. Dev.

[b22] Qian H, Hu B, Cao D, Chen W, Xu X, Lu Y (2007). Bio-safety assessment of validamycin formulation on bacterial and fungal biomass in soil monitored by real-time PCR. Bull. Environ. Contam. Toxicol.

[b23] Schimel J, Balser TC, Wallenstein M (2007). Microbial stress-response physiology and its implications for ecosystem function. Ecology.

[b24] Singh BK, Bardgett RD, Smith P, Reay DS (2010). Microorganisms and climate change: terrestrial feedbacks and mitigation options. Nat. Rev. Microbiol.

[b25] Sofo A, Palese AM, Casacchia T, Celano G, Ricciuti P, Curci M, Crecchio C, Xiloyannis C (2010). Genetic, functional, and metabolic responses of soil microbiota in a sustainable olive orchard. Soil Sci.

[b26] Stromberger ME, Shah Z, Westfall DG (2011). High specific activity in low microbial biomass soils across a no-till evapotranspiration gradient in Colorado. Soil Biol. Biochem.

[b27] Torsvik V, Øvreås L (2002). Microbial diversity and function in soil: from genes to ecosystems. Curr. Opin. Microbiol.

